# Resected case of stage IV pleomorphic carcinoma of the lung with long-term survival

**DOI:** 10.1186/s40792-020-00868-z

**Published:** 2020-05-24

**Authors:** Haruki Choshi, Mototsugu Watanabe, Hiroyuki Ujike, Yumiko Sato, Toshiaki Morito, Ryujiro Sugimoto, Kazuhiko Kataoka

**Affiliations:** 1 Department of Thoracic Surgery, Iwakuni Clinical Center, 1-1-1 Atago-machi, Iwakuni-shi, Yamaguchi, 740-8510 Japan; 2 Department of Diagnostic Pathology, Iwakuni Clinical Center, Yamaguchi, Japan; 3 Department of Cardiovascular and Thoracic Surgery, Ehime University Graduate School of Medicine, 454 Shitsukawa, Touon-shi, Ehime Japan

**Keywords:** Pleomorphic carcinoma, Oligometastasis, Adrenal hemorrhage

## Abstract

**Background:**

No established treatments for pulmonary pleomorphic carcinoma exist because of its rarity, and the prognosis is poorer than that of other non-small cell lung cancers.

**Case report:**

We present a case of stage IV pleomorphic carcinoma; the patient was a 66-year-old male. He was referred to our hospital because of a right adrenal hemorrhage and a lung tumor. A systemic examination revealed that the lung tumor was a primary lung cancer and that the adrenal hemorrhage was due to a metastatic cancer. We performed an adrenalectomy and resection of the lung tumor and obtained a diagnosis of pleomorphic carcinoma with adrenal metastasis. The patient has remained recurrence-free for 6 years since the surgery.

**Conclusions:**

We report a patient with stage IV pleomorphic carcinoma of the lung and an oligometastasis in whom a complete resection enabled a good outcome. Additional reports are needed to clarify definite prognostic factors and the optimal treatment for pleomorphic carcinoma.

## Background

Pleomorphic carcinoma is a rare type of non-small cell lung cancer (NSCLC) that accounts for approximately 0.1–0.4% of primary lung tumors [1]. No established treatments exist because of its rarity; consequently, pleomorphic carcinoma is associated with a worse outcome than other NSCLC. In addition, the presence of distant metastases can worsen the prognosis [1–3]. Although the adrenal gland is a common organ for distant metastases of primary lung cancer [4], adrenal metastases are often asymptomatic. Adrenal hemorrhages are relative rare, can cause pain, and can worsen the prognosis because of circulatory failure, an adrenal crisis, or other events [5–8].

In this report, we described a rare patient with pleomorphic carcinoma and adrenal hemorrhage who survived without any recurrences for more than 6 years after a complete resection.

## Case presentation

A 66-year-old man was admitted to the clinic of a primary care doctor complaining of a sudden deterioration in right hypochondoralgia persisting for 2 weeks. A computed tomography (CT) scan revealed a right adrenal hemorrhage and an abnormal tumor in the upper lobe of his left lung. He was subsequently referred to our hospital for further examination and treatment.

**Fig. 1:**
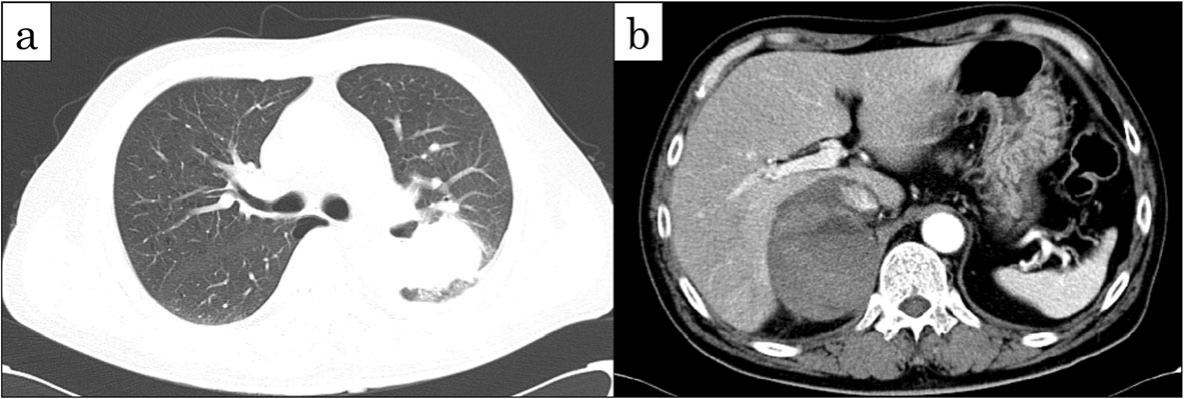
CT scan showed abnormal masses. **a** Chest CT scan showed a 42-mm tumor in the left lung S1 + 2 progressing to S6. **b** Abdominal enhanced CT scan showed a 82-mm mass with no active bleeding

His past medical history and family history were unremarkable. He was a current smoker with a history of 46 pack-years. An enhanced CT scan showed a massive shadow in the left lung S1 + 2 progressing to S6 beyond the lung lobe, with a maximum diameter of about 42 mm (Fig. 1a), and a right adrenal hematoma with no active bleeding (Fig. 1b). The laboratory data revealed a slight elevation in carcinoembryonic antigen (CEA, 5.6 ng/mL) and neuron-specific enolase (NSE, 18.52 ng/mL). The patient had mild anemia (hemoglobin, 10.3 g/dL).

**Fig. 2:**
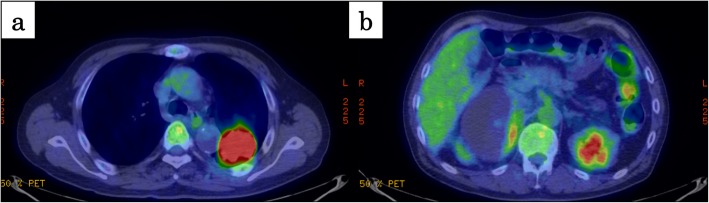
Imaging of FDG-PET. **a** There was high accumulation of FDG at the left lung tumor (SUVmax, 17.0). **b** There was slightly accumulation of FDG at the edge of abdominal tumor (SUVmax, 4.1)

A diagnosis of the pulmonary lesion using a bronchoscopic trans-bronchial lung biopsy showed no evidence of malignancy; therefore, a CT-guided percutaneous needle biopsy was performed. The pathological examination showed a non-small cell lung cancer (NSCLC) that was suspected to be a pleomorphic carcinoma. The adrenal lesion was diagnosed as a nonfunctional tumor based on endocrine examinations and adrenal medulla scintigraphy (^123^I-MIBG). 18-Fluorodeoxyglucose positron emission tomography (FDG-PET) showed the accumulation of FDG not only in the left lung nodule (SUVmax, 17.0) (Fig. 2a) but also in the right adrenal one (SUVmax, 4.1) (Fig. 2b). Together, these results suggested a diagnosis of NSCLC and adrenal metastasis, and the clinical stage was classified as cT2bN0M1b, stage IV (TNM classification 7th edition).

**Fig. 3:**
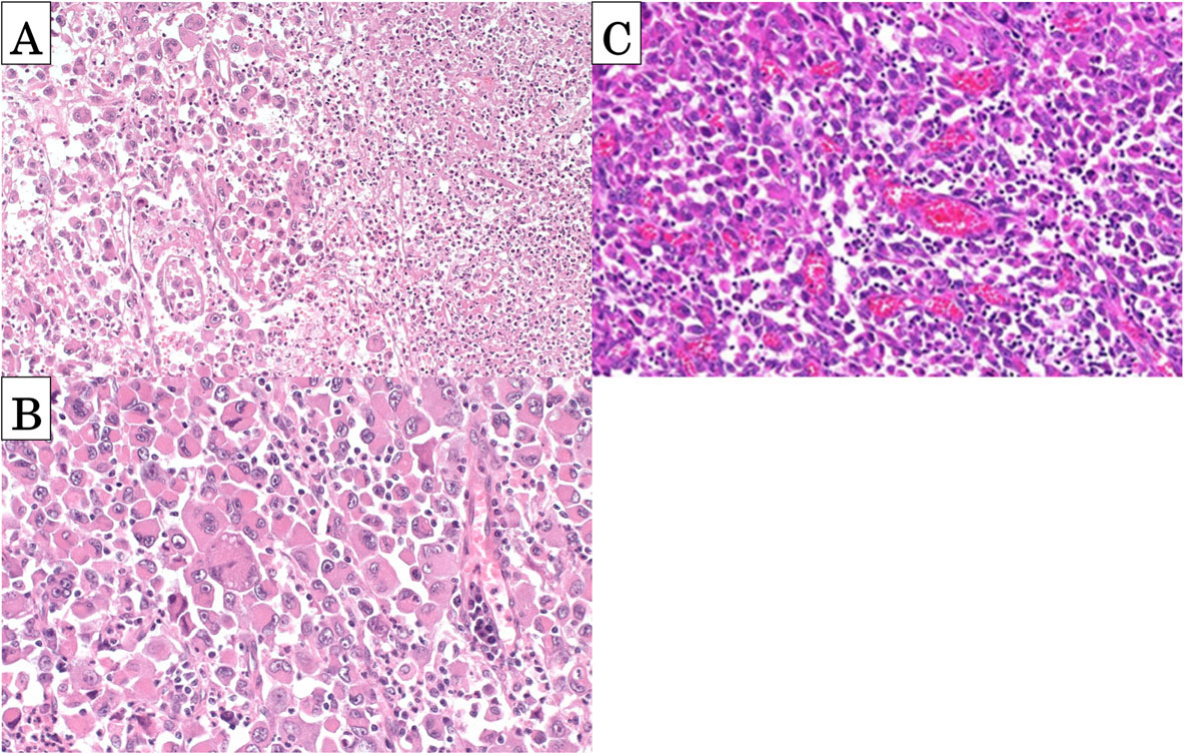
The pathologic analysis. **a**, **b** Pathologic analysis of the lung tumor revealed atypical giant cells with eosinophilic cytoplasms as well as sarcomatous component with necrosis. **c** Pathologic analysis of the adrenal tumor revealed similar histologic appearance to lung tumor

Because there was a risk of rebleeding from adrenal metastasis, we performed a right adrenalectomy. We considered that a complete resection of the primary lung cancer would be possible since the adrenal metastasis was an oligometastasis. A pathological analysis of the lung tumor demonstrated areas of atypical cells with eosinophilic cytoplasms as well as sarcomatous component, with extensive necrosis (Fig. 3a, b). Immunohistochemistry revealed that the tumor cells were positive for cytokeratin AE1/3, CAM5.2, CK7, and p63 (partial) but negative for 34βE12, CK20, TTF-1, calretinin, and D2-40. The adrenal tumor was similar in pathologic and immunohistochemical analyses (Fig. 3c). Thus, the final diagnosis was pleomorphic carcinoma of the lung with an adrenal metastasis, pT2bN0M1b, stage IV (TNM classification 7th edition).

The patient received chemotherapy as stage IV NSCLC (60 mg/m^2^ of cisplatin on day 1 and 40 mg/m^2^ of TS-1 twice a day on days 1 to 14, repeated every 4 weeks). After two courses, he decided to quit the intravenous chemotherapy because of adverse events, general malaise, and anorexia. We suggested to receive oral chemotherapy, but he refused to take TS-1 because he feared adverse events. Consequently, he began taking UFT (600 mg/day) and continued this treatment for 2 years. He has survived without any recurrences for 6 years since the surgery.

## Discussion

Pleomorphic carcinoma is a rare type of NSCLC and has been reported to account for 0.1 to 0.4% of primary lung tumors [1]. The mean age at diagnosis was 60 to 65 years. The male to female ratio is 2.1:1, and 60 to 90% of all patients are smokers. Because the upper lobe is more frequently in contact with cigarette smoke, the upper lobe (48–69%), particularly the right upper lobe (33–50%), is the most common site [1, 9, 10].

In the 2015 World Health Organization (WHO) histologic classification of lung tumors, pleomorphic carcinoma was classified as a subtype of sarcomatoid carcinoma. Sarcomatoid carcinoma is grouped into pleomorphic carcinoma, spindle cell carcinoma, giant cell carcinoma, carcinosarcoma, and pulmonary blastoma. A diagnosis of pleomorphic carcinoma cannot be made without a complete evaluation of the entire tumor histologically. Pleomorphic carcinoma consists of poorly differentiated adenocarcinoma, squamous cell carcinoma, or large cell carcinoma containing at least 10% spindle and/or giant cells or a carcinoma comprised only of spindle and giant cells [11]. For tumors containing a wide range of cellular components, pleomorphic carcinoma is the most common subtype of sarcomatoid carcinomas [12].

In general, patients with pleomorphic carcinoma received same treatment with other NSCLCs. However, an effective chemotherapy for pleomorphic carcinoma has not been established because of the insufficient data on pleomorphic carcinoma, particularly a lack of long-term follow-up data. Previous studies reported that the overall survival rate was worse than those of other NSCLCs, with 1- and 5-year values of 45.5% and 20.1%, respectively. The reported prognostic factors were tumor size (> 5–7 cm), presence of distant or lymph node metastasis, and a clinical stage of > I, among which lymph node metastasis has attracted attention [12–16]. Stage IV pleomorphic carcinoma typically contains most of these factors, resulting in a remarkably low 5-year survival rate. Most studies focused on lymph node metastases have compared pN0 and pN2 without distant metastasis [16–18]; thus, the prognostic factors for stage IV pleomorphic carcinoma have not been studied.

However, there are some reports of stage IV pleomorphic carcinoma cases obtaining long-term survival after resections. To our knowledge, five cases have been reported, and the features of these cases are shown in Table 1 [1, 3, 19–21]. None of these cases had lymph node metastasis. The cases shown in Table 1 suggest that patients with limited distant metastasis can obtain better outcomes after a complete primary lung tumor resection with metastasectomy if they do not have lymph node metastasis. Complete resection may contribute to the long-term survival of patients with stage IV pleomorphic carcinoma. In addition, Yokoyama et al. reported that the presence of tumor spread through air spaces (STAS) was a useful prognostic factor among cases of surgically resected lung pleomorphic carcinoma [22]. Most cases in the recent report were of stages other than stage IV because all the cases had undergone resection. Further studies in stage IV patients are needed. In our case, the adrenal gland was the only site of metastasis, and no lymph node metastasis was seen; consequently, we were able to perform a complete resection and to obtain long-term survival.

**Table 1:** The features of stage IV pleomorphic carcinoma cases getting long-term survival after resections

	Case 1	Case 2	Case 3	Case 4	Case 5	Case 6
Age	71	48	63	69	62	66
Sex	Male	Male	Male	Male	Male	Male
Primary lesion	Right lower lobe	Right upper lobe	Left upper lobe	Right upper lobe	Left upper lobe	Left upper lobe
Size (mm)	70	65	73	48	33	42
Metastasis lesion	Brain	Jejunum	Small bowel	Stomach	Stomach	Right adrenal grand
Lymph node metastasis	No	No	No	No	No	No
Chemotherapy	Induction	No	Adjuvant	No	No	Adjuvant
	CBDCA+ GEM		CBDCA+ GEM			CDDP+ S-1
	6 courses					→ UFT
Recurrence-free time (year)	7	6	1	5	4	6

In addition to the progression of cancer itself, our case experienced an adrenal hemorrhage secondary to the adrenal metastasis, which is associated with a high mortality rate. In general, most adrenal metastases are asymptomatic and are often detected incidentally by CT scan. Almost 40% of NSCLC patients develop adrenal metastases [6, 7, 23]. An analysis adjusted according to blood flow distribution reported that lung cancer metastasized to the adrenal gland most frequently [4]. In contrast, adrenal metastases-related hemorrhages are rare in patients with primary lung cancer and cause acute pain. To our knowledge, only 30 cases have been reported worldwide, including the present case [5–8]. Among the clinical features, 28 cases experienced pain, 18 cases had anemia, and 10 cases had nausea or vomiting. Regarding the pathological diagnosis of the primary lung cancer, many cases were adenocarcinoma (*n* = 12), followed by large cell carcinoma (*n* = 7) and pleomorphic carcinoma (*n* = 3, 10%). The prognosis of adrenal metastasis-related hemorrhage is poor. Survival for more than 1 year has only been confirmed in three cases. The most common cause of death was hemorrhage (*n* = 10), followed by cancer progression (*n* = 4) and infection (*n* = 3) [6, 8]. The control of these factors can allow long-term survival for patients with adrenal hemorrhages. In our case, the adrenal lesion was a hematoma with no active bleeding. Because there was a risk of rebleeding, we performed a right adrenalectomy to control the disease. Additionally, the resection of the primary site was considered optimal, as described above. We performed a complete resection for our case, and the patient obtained a long-term survival.

Our case suggests that a complete resection can enable long-term survival in patients with stage IV pleomorphic carcinoma and adrenal hemorrhage. Because few stage IV cases with long-term follow-up have been reported, further cases should be gathered and examined to establish an optimal treatment for pleomorphic carcinoma.

## Conclusion

We report a rare case of pleomorphic carcinoma that was discovered incidentally to adrenal bleeding; surgery was possible, and long-term survival was obtained. Complete resection may enable a good outcome in patients with stage IV pleomorphic carcinoma of the lung with adrenal hemorrhage secondary to adrenal metastasis. Further studies are warranted to provide prognostic factors and surgical benefits for this poor prognostic disease.

## Data Availability

Not applicable

## Abbreviations

NSCLC: Non-small cell lung cancer
CT: Computed tomography
CEA: Carcinoembryonic antigen
NSE: Neuron-specific enolase
FDG-PET: 18-Fluorodeoxyglucose positron emission tomography
WHO: World Health Organization
STAS: Spread through air spaces
